# Identification of cytochrome c oxidase subunit 4 isoform 1 as a positive regulator of influenza virus replication

**DOI:** 10.3389/fmicb.2022.862205

**Published:** 2022-07-19

**Authors:** Jun He, Huibin Huang, Bo Li, Huanan Li, Yue Zhao, Yaolan Li, Wencai Ye, Wenbao Qi, Wei Tang, Lei Wang

**Affiliations:** ^1^Center for Bioactive Natural Molecules and Innovative Drugs Research, Guangdong Province Key Laboratory of Pharmacodynamic Constituents of TCM and New Drugs Research, College of Pharmacy, Jinan University, Guangzhou, China; ^2^Institute of Laboratory Animal Science, Jinan University, Guangzhou, China; ^3^Pharmacy Department, Wenzhou People’s Hospital, Wenzhou, China; ^4^National Avian Influenza Professional Laboratory, Key Laboratory of Zoonoses, Ministry of Agriculture, South China Agricultural University, Guangzhou, China; ^5^Chongqing Academy of Animal Sciences, Chongqing, China

**Keywords:** highly pathogenic H5N1 influenza virus, lycorine, anti-influenza, viral ribonucleoproteins export, COX41

## Abstract

Human infection with highly pathogenic H5N1 influenza virus causes severe respiratory diseases. Currently, the drugs against H5N1 are limited to virus-targeted inhibitors. However, drug resistance caused by these inhibitors is becoming a serious threat to global public health. An alternative strategy to reduce the resistance risk is to develop antiviral drugs targeting host cell proteins. In this study, we demonstrated that cytochrome c oxidase subunit 4 isoform 1 (COX41) of host cell plays an important role in H5N1 infection. Overexpression of COX41 promoted viral replication, which was inhibited by silencing or knockout the expression of COX41 in the host cell. The ribonucleoproteins (RNPs) of H5N1 were retained in the cell nucleus after knockout cellular COX41. Strikingly, inhibition of cellular COX41 by lycorine, a small-molecule compound isolated from Amaryllidaceae plants, reduced the levels of COX41-induced ROS and phosphorylation of extracellular signal-regulated kinase (ERK) in cells, thus resulting in the blockage of nuclear export of vRNP and inhibition of viral replication. In H5N1-infected mice that were treated with lycorine, we observed a reduction of viral titers and inhibition of pathological changes in the lung and trachea tissues. Importantly, no resistant virus was generated after culturing the virus with the continuous treatment of lycorine. Collectively, these findings suggest that COX41 is a positive regulator of H5N1 replication and might serve as an alternative target for anti-influenza drug development.

## Introduction

The highly pathogenic influenza viruses, such as H5N1, occasionally infect humans and cause severe cute respiratory diseases, leading to an enormous impact on the global economy and public health (Creanga et al., 2017). Vaccine and small molecule drugs are currently two main treatment strategies for influenza virus infection. Because an effective vaccine usually takes at least 6 months to develop and has limited efficacy on immunocompromised patients, the small-molecule drugs have become the first line of protection against the epidemic outbreak at the early stage (Shen et al., 2015). Up to now, most of the drugs against influenza virus target the viral proteins, such as the inhibitors of viral matrix 2 (M2), neuraminidase (e.g., amantadine and oseltamivir), and polymerase acidic (PA) proteins (baloxavir; Saelens, 2019; Jalily et al., 2020). However, the mutant viruses conferring resistance to these inhibitors are generated due to the high mutation rate of the viral genome, leading to the loss of their therapeutic efficacy in the clinic (Holmes et al., 2021). Statistically, most of the globally circulating AIV H1N1 subtypes are resistant to oseltamivir until the H1N1 outbreak in 2009 (Ledesma et al., 2011). More seriously, mutations in the viral hemagglutinin (HA) or neuraminidase (NA) proteins that are elicited by oseltamivir can result in the escape of the mutant virus from host immune surveillance, which may favor the virus transmission in the population (Petrova and Russell, 2018). Therefore, the development of alternative anti-influenza drugs is urgently needed. Searching for host-targeted inhibitors is considered as a realistic strategy to reduce the risk of drug resistance.

Cytochrome c oxidase (CcO), composed of 3 mitochondrial DNA-encoded subunits and 10 nuclear-encoded subunits, is the terminal enzyme of the mitochondrial respiratory chain, which catalyzes the final step of electron transfer from cytochrome c (cyt c) to oxygen (O_2_; Srinivasan and Avadhani, 2012). Nuclear-encoded cytochrome c oxidase subunit 4 (COX4) is a key regulatory subunit of mammalian CcO, which is formed by cytochrome c oxidase subunit 4 isoform 1 (COX41) and COX4 isoform 2 (COX42; Bikas et al., 2020). The principal isoform COX41 has been demonstrated to be associated with glioma chemoresistance and mitochondrial disorders (Oliva et al., 2017; Douiev et al., 2021). However, the function of COX41 involved in viral replication is rarely reported yet.

Lycorine, as the main component of *Lycoris radiata,* can inhibit several viral species, such as poliovirus, severe acute respiratory syndrome-associated coronavirus, human enterovirus 71, hepatitis C virus, HIV-1, herpes simplex virus, and yellow fever virus (Zou et al., 2009; Guo et al., 2016; Prinsloo et al., 2018; Shen et al., 2019; Verma et al., 2020). However, the molecular mechanism of lycorine on viral infections is still ambiguous. In our previous study, we have reported that lycorine inhibited the infections of different subtypes of the influenza virus in Madin Darby Canine Kidney (MDCK) cells, in which the concentration for 90% maximal effect (EC_90_) of lycorine was lower than that of oseltamivir. Moreover, our results have demonstrated that lycorine prevented the nuclear export of the viral ribonucleoprotein (vRNP) complexes (He et al., 2013).

In this study, we found that the replication of H5N1 was positively regulated by cellular COX41. Notably, lycorine reduced the levels of COX41 in cells and blocked the nuclear export of vRNP. In H5N1-infected mice that were treated with lycorine, reduced pathologic changes and viral loads were detected in the lung and trachea tissues of mice. These results provide direct evidence of the positive correlation between cellular COX41 levels and influenza virus infection. Inhibition of COX41 in the host cell may serve as a viable approach for anti-influenza therapy.

## Materials and methods

### Virus, cells, and antibodies

The H5N1 influenza virus strain chicken/Guangdong/178/2004 (GD178, GenBank Accession No. AY737296–737300), MDCK cells, and human embryonic kidney 293 (HEK293) cells were obtained from the National and Regional Joint Engineering Laboratory for Medicament of Zoonosis Prevention and Control in China or purchased from the China Center for Type Culture Collection. All of the cells were grown in DMEM (Invitrogen, Carlsbad, CA, United States) supplemented with 10% (v v^−1^) FBS (Invitrogen). Viral titers were determined by plaque assay or 50% tissue culture infective dose (TCID_50_) in MDCK cells (He et al., 2013). All infection experiments were performed in BSL-3 facilities. Lycorine was isolated as previously described (He et al., 2013).

The antibodies used were as follows: anti-COX41 (ab66739, Abcam, United Kingdom), anti-ERK1/2 (4695P, CST, United States), anti-phospho-ERK1/2 (4370P, CST, United States), anti-Akt (9,272, CST, United States), anti-Phospho-Akt (Ser473; 4,060, CST, United States), anti-avian influenza virus (AIV) nucleoprotein (NP; Perfect Biotechnology, China), and anti-β-actin (20536-I-AP, Proteintech, United States).

### Plaque assay

The plaque assay was adapted from a previously described procedure (Tian et al., 2012; Tang et al., 2021). MDCK cells grown in 6-well plates were inoculated with virus suspensions for 1 h at 37°C. Afterward, the cells were washed twice with PBS and overlaid with 1% agarose in MEM (Wang et al., 2018). After 48–72 h of incubation at 37°C, the agarose in each well was removed. The cells were stained with 0.5% crystal violet in 10% formaldehyde solution. After washing with PBS, the virus-induced plaques were visualized and counted.

### Western blot assay

Equal amounts of proteins were separated using SDS-polyacrylamide gel electrophoresis (SDS-PAGE) and subsequently transferred to PVDF membranes (Bio-Rad). The membranes were blocked with 5% BSA for 1 h and then incubated with primary antibodies at 4°C overnight. After washing twice with TBST, the membranes were incubated with horseradish peroxidase (HRP)-conjugated secondary antibody (1:2,000; 7074P CST, United States) for 2 h at room temperature. Pierce ECL Western blot substrate (Thermo Fisher Scientific, United States) was used to visualize specific protein bands, and the band intensity was quantified using ImageJ software.

### Proteomic analysis

SILAC was conducted using the mock-treated control (L), influenza virus-infected cells (M), and lycorine-treated cells (H), respectively. MDCK cells were infected with H5N1 in the presence or absence of lycorine. At 12 h after infection, the cells were harvested and lysed in SDT buffer [4% (w v^−1^) SDS, 150 mM Tris/HCl, pH 7.4]. After boiling for 7 min, the cell lysates were centrifuged at 12,000 × *g* for 15 min at 4°C. The total proteins from three groups were mixed equally, separated by 1D SDS-PAGE, and stained with Coomassie Brilliant Blue. Then, the entire gel lanes were cut into 10 slices and each slice was destained with 30% ACN∙100 mM^−1^ NH_4_HCO_3_. The dried gels were treated with 10 mM DTT for 40 min at 55°C and then alkylated with iodoacetamide buffer (200 mM IAA∙100 mM^−1^ NH_4_HCO_3_) for 30 min. The gels were gently washed with 100 mM NH_4_HCO_3_ and ACN and digested using 12.5 ng μl ^−1^ trypsin in 25 mM NH_4_HCO_3._ The peptides were extracted with 60% ACN/0.1% TFA, dried through a vacuum centrifuge, and then analyzed with a Q Exactive mass spectrometer coupled to an Easy-nLC system for 60 min (Proxeon Biosystems, now Thermo Fisher Scientific). Peptides from each sample (10 μl) were subjected to nano-LC–MS/MS analysis and identified using MaxQuant software, version 1.3.0.5.

### Detection of COX41 expression

The *COX4I1* genes were amplified and subcloned into a CMV-FLAG-tagged vector (Beyotime, China). The recombinant plasmids were transfected with PEI (Sigma) into HEK 293 T cells. At 4 h after transfection, the culture medium was replaced with Opti-MEM. After incubation for 24 h, the cells were collected and subjected to RT-PCR and western blot analysis.

### Knockdown of COX41

Specific siRNAs targeting *COX4I1* genes (si-COX41-1, si-COX41-2, and si-COX41-3) and non-specific siRNA (scrambled siRNA) were synthesized by RiboBio Co., Ltd. HEK 293 T cells in 6-well plates were transfected with siRNAs (50 nm) in 20 μl of Lipofectamine 2000 (Invitrogen) and 1 ml of Opti-MEM. The supernatants were removed after 4 h of transfection and supplemented with cell culture medium. At 48 h after transfection, the cells were collected and subjected to PT-PCR or western blot analysis.

### CRISPR/Cas9 plasmids and generation of COX41 knockout cells

The oligonucleotide sequences used to generate single guide RNAs (sgRNAs) were selected from a sgRNA database published by the Feng Zhang Laboratory^1^ as follows: COX41-1, Forward: 5¢-CACCGTCACCGCGCTCGTTATCATG-3¢, Reverse: 5¢-AAACC ATGATAACGAGCGCGGTGAC-3¢; COX41-2, Forward: 5¢-CACCGCTGCCACATGATAACGAGCG-3¢, Reverse: 5¢-AAACCGCTCGTTATCATGTGGCAGC-3¢. These sgRNAs were synthesized by the Beijing Genomics Institute (BGI, China). To clone the sgRNA guide sequence, plasmids were cut and dephosphorylated at 37°C for 2 h. The COX41 sgRNA guide sequences were phosphorylated using polynucleotide kinase (Fermentas) for 30 min at 37°C, annealed by heating to 95°C for 5 min, cooled at 25°C, and then ligated into lentiCRISPR v2 (Addgene; plasmid 52,961) at 25°C for 5 min by using T7 ligase (Enzymatics; Sanjana et al., 2014). To construct the lentivirus, transfer vectors were co-transfected with the pMD2.G (Addgene; plasmid 12,259) and psPAX2 (Addgene; plasmid 12260). Briefly, HEK 293 T cells were transfected with transfer vector, pMD2.G, and psPAX2 plasmids using Lipofectamine 2000 (Life Technologies). At 6 h after transfection, the culture medium was replaced with DMEM containing 10% FBS. After incubation for 60 h, the viral suspensions were collected and stored at −80°C until use. HEK293T cells were infected with the constructed lentivirus for 48 h and treated with puromycin (1 μg ml^−1^) for 12 days. PCR was then performed using the primer pair CRISPR-1, Forward: 5¢-CCGGAATTCATGCATAGTGTTTGGTATG-3¢, Reverse: 5¢-CCGCTC GAGTCAATGAATGAGTCCCCTT-3¢. The level of COX41 in cells was determined by Western blot assay.

### Intracellular vRNP localization

HEK293T cells and KO-COX41 HEK293T cells were infected with the GD178 virus for 1 h, respectively. The cell supernatants were then removed and replaced. At 12 h or 24 h after infection, the cells were fixed with 4% paraformaldehyde, washed, and stained with anti-NP antibody (Perfect Biotechnology, Beijing, China) and Alexa Fluor (488 nm) secondary antibody (Zhongshan Golden Bridge Biotechnology, Beijing, China). Afterward, the cells were stained with DAPI and photographed using a confocal laser scanning microscope. Mean fluorescence intensity associated with expression level of NP was analyzed by Image J software.

### Viral growth kinetics

Confluent wild-type (WT) cells, COX41-KO-1 cells, and COX41-KO-1 cells with transfection of plasmids expressing Flag-COX41 were infected with the GD178 virus at an MOI of 0.01. Viral inoculant was removed after 1 h of adsorption at 37°C and replaced with DMEM. Infected cells were then incubated at 37°C in 5% CO_2_. Virus suspensions were collected at 12, 24, 36, 48, and 72 h postinfection (h.p.i), respectively. Viral titers were determined by the standard TCID_50_ assay in MDCK cells.

### ROS assay

Cells in the catalase (CAT) control group were treated with 1 mg ml^−1^ CAT (Sigma, St. Louis, MO, United States) for 1 h. After adsorption of 0.01 MOI virus for 1 h, different concentrations of lycorine were added, and the cells were washed twice with PBS and then stained with 10 μM 2′,7′-dichlorofluorescein diacetate (Sigma, St. Louis, MO, United States) in PBS for 30 min in the dark for different time periods. The cells were then washed twice with PBS. Fluorescence was recorded using a spectrofluorometer (Thermo Fisher Scientific, United States) with an excitation wavelength of 490 nm and an emission wavelength of 525 nm.

### Studies in mice

BALB/c mice (female, 6–8 weeks old, 16–18 g) were purchased from Beijing Vital River Laboratory Animal Technology Co., Ltd. (Beijing HFK Bioscience Co. Ltd., China) and bred in BSL-3 facilities at South China Agriculture University (China). This animal strain is widely used in the study of AIV infection (Sang et al., 2021; Wu et al., 2021). The mice were housed under controlled temperature, humidity, and lighting (23°C ± 1°C; 12: 12 h light–dark cycle) conditions and provided with free access to food and sterile water. In the infection experiments, the mice were anesthetized with isoflurane and placed in a rodent anesthesia machine (R540; RWD Life Science Co., Ltd., China). Isoflurane (4%) was delivered by a nose cone for 2–3 min. The experimenters were blinded to group assignment and outcome assessment.

The mice were allowed to adapt to their housing environment for at least 7 days before experimentation. After a 7-day quarantine period, mice were administered with 5 mg kg^−1^ day^−1^ lycorine or mock (PBS with the same concentration of DMSO as the lycorine-treated groups) by i.p. injection at 1 day prior to viral infection (2 LD_50_) for 16 consecutive days. On day 5 or 14 postinfection, the mice were euthanized and lungs were collected for subsequent experiments.

### Viral titers and lung index

The mice were euthanized on day 5 after viral infection using pentobarbital sodium (200 mg kg^−1^). The lungs of mice were collected and weighed. The lung index was determined as follows: Lung index = lung weight (g)/body weight (g) × 100 (Myers et al., 2021). Each lung was homogenized in 1 ml of DMEM with 100 U/ml penicillin/streptomycin (Invitrogen, Carlsbad, CA, United States) and centrifuged at 3,000 × *g* for 5 min at 4°C. The supernatants of lung homogenates were then harvested and subjected to viral titer determination (Tang et al., 2019).

### Histopathological analyses of lung and trachea tissues

Specimens from the right lung and trachea from each group were fixed in 4% paraformaldehyde for 24–48 h. The fixed tissue samples were dehydrated by using a graded ethanol series and then embedded in paraffin. For hematoxylin–eosin (H&E) staining, 4 μm-thick serial sections were prepared using routine protocols and observed at 400 × magnification (Leica DM6000).

### Quantitative real-time PCR

Total RNA in cells or lung tissues of mice was extracted according to the manufacturer’s instructions (RNAfast200, Fastagen Biotech) and reverse transcribed into cDNA using a mixture of random and oligo (dT) primers. The primers for the amplification of *COX4I1* genes were designed as follows: canine *COX4I1* (132 bp), Forward: 5′-CTTTATCGGCTTCACTGCT-3′, Reverse: 5′-CATGAAAGTCAGCCCGATT-3′; human *COX4I1* (108 bp), Forward: 5′-ATGTCAAGCACCTGTCTGC-3′, Reverse: 5′-TGAACTTAATGCGATACAACTC-3′. RT-qPCR assays were conducted using a LightCycler 480 system (Roche, Germany) as follows: 94°C for 5 min, 30 cycles of 94°C for 10 s, 53°C for 10 s, and 72°C for 20 s, and 72°C for 10 min. The cellular GAPDH genes were used as a positive control to assess cDNA quality.

### Statistical analyses

All data were expressed as the mean ± standard deviation (SD). Student’s *t*-test was used to identify statistically significant differences between groups. *p* values ≤ 0.05 were considered a statistically significant difference between compared groups. Data were analyzed with GraphPad Prism software, version 8.0 (GraphPad Software Inc., La Jolla, CA, United States).

## Results

### COX41 involves in the lifecycle of H5N1

**Figure 1:**
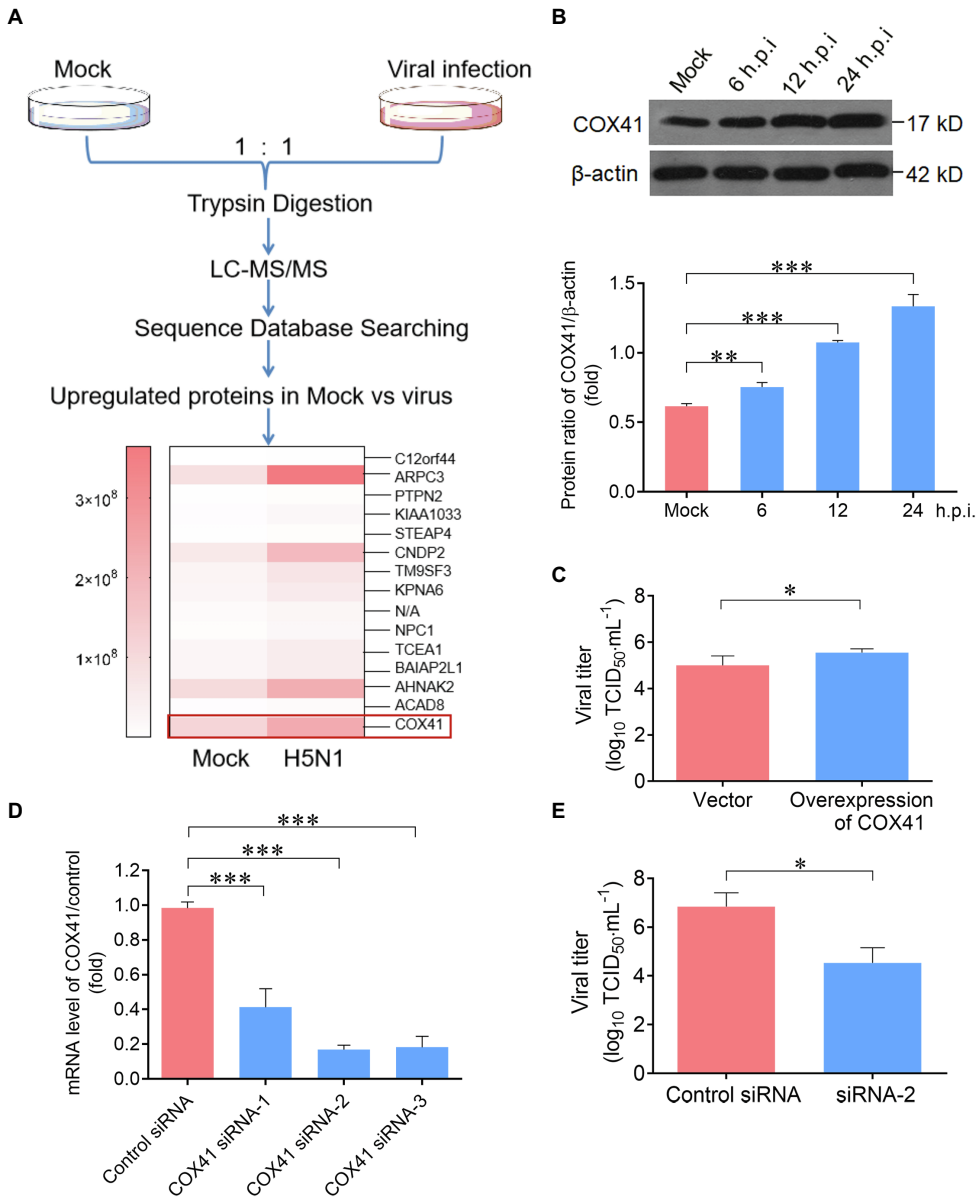
Cytochrome c oxidase subunit 4 isoform 1 (COX41) was involved in the replication of H5N1. **(A)** Scheme illustrating the proteomic analysis of Madin Darby Canine Kidney (MDCK) cells in responses to H5N1 infection. MDCK cells were infected with H5N1 (MOI, 1) and detected at 12 h. The differentially expressed proteins were presented as a heat map. **(B)** Expression of COX41 was upregulated by H5N1 infection. MDCK cells were infected with H5N1 for 6 h (MOI, 5), 12 h (MOI, 5), and 24 h (MOI, 0.01), respectively. The level of COX41 was assessed by Western blot and quantitatively analyzed using ImageJ software. Data are mean ± s.d. (*n* = 2 ~ 3). ^**^p < 0.01; ^***^p < 0.001. **(C)** Overexpression of COX41 in cells increased the viral titers. HEK293T cells were transfected with plasmids expressing FLAG-tagged COX41 and then infected with H5N1 (MOI, 0.01). Viral titers were assessed at 72 h postinfection by TCID_50_ assay. Data are mean ± s.d. (*n* = 3). ^*^p < 0.05. **(D)** Detection of COX41 mRNA level in HEK293T cells transfected with COX41 siRNAs targeting COX41 genes at 24 h.p.i. Data are mean ± s.d. (*n* = 3). **(E)** Effects of COX41 siRNAs on viral titers as determined by TCID_50_ assays. HEK 293 T cells were transfected with siRNAs and then infected with H5N1. The viral titers in cells were determined at 48 h.p.i. Data are mean ± s.d. (*n* = 3). ^*^p < 0.05; ^**^p < 0.01; ^***^p < 0.001.

To comprehensively understand the protein profile of H5N1 (GD178 strains) infection, stable-isotope labeling by amino acids in cell culture (SILAC) proteomics method was applied. As shown in Figure 1A, the expression level of COX41 protein in H5N1-infected cells was greatly increased as compared to that of uninfected cells (mock). This result has attracted our attention because the function of COX41 is to generate ROS, which has an important role in influenza virus infection (Vlahos et al., 2011; Lin et al., 2016; Pajuelo Reguera et al., 2020).

Next, three sets of experiments were performed to investigate the effects of cellular COX41 on H5N1 replication. First, the expression levels of COX41 in cells were determined at different time points (single or multiple replication cycles) after the viral infection. Consistent with the results of SILAC and western blot experiments, the expression of COX41 in cells was substantially upregulated after H5N1 infection. At 24 h postinfection (h.p.i.), the level of COX41 in H5N1-infected cells was increased nearly 2-fold compared with uninfected cells (mock; Figure 1B). Second, the viral titers in cells after transfection with COX41 plasmids were measured by TCID_50_ assay. Overexpression of COX41 significantly increased the viral titers by approximately 2.5-fold at 72 h postinfection (Figure 1C). Third, the viral titers in cells after transfection of COX41 siRNAs were significantly reduced as compared with the cells transfected control siRNA (Figures 1D,E). These results suggest that the replication of H5N1 is positively regulated by COX41.

### Knockout of COX41 expression blocks vRNP export

**Figure 2:**
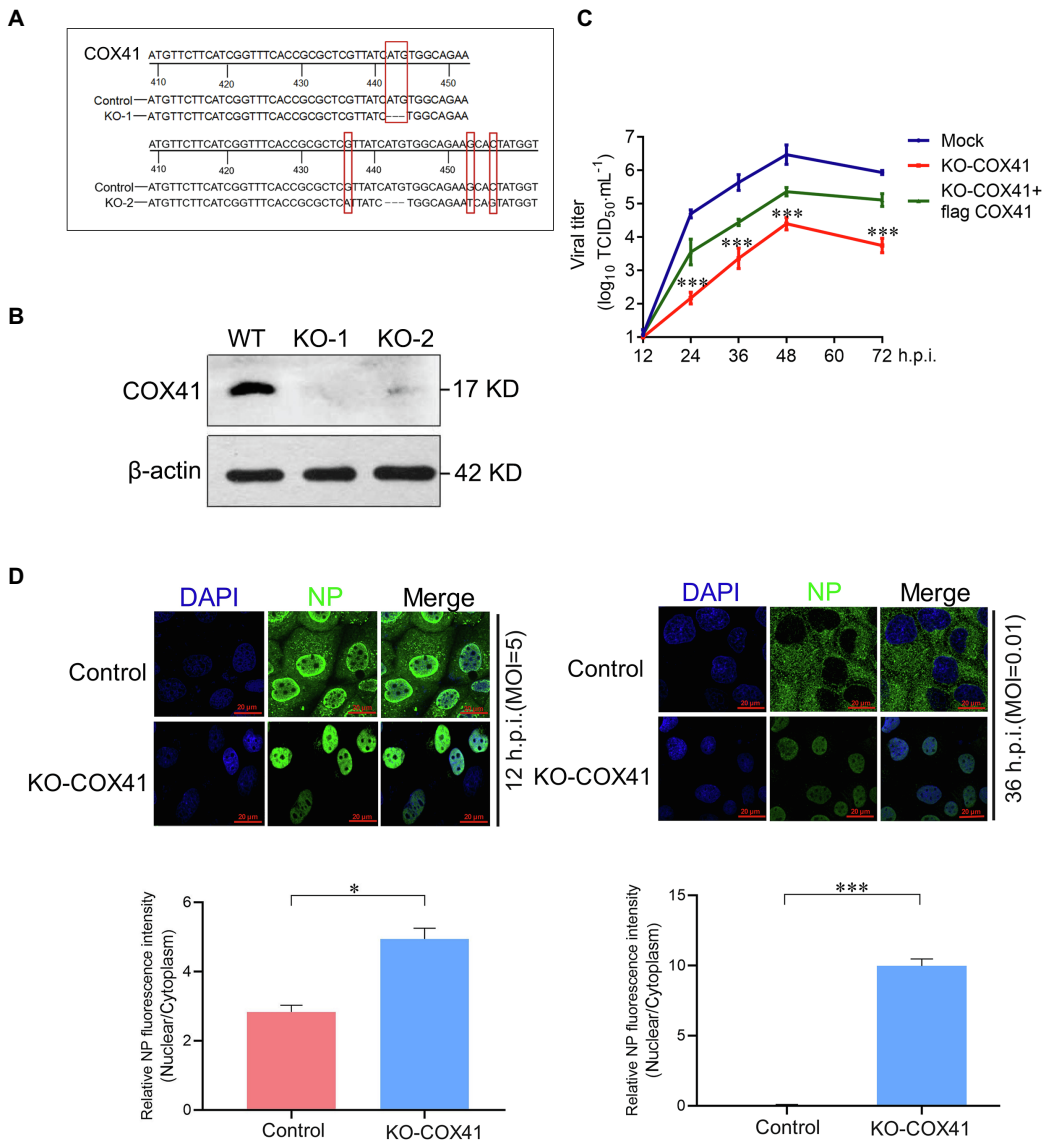
Cytochrome c oxidase subunit 4 isoform 1 (COX41) was involved in the nuclear export of vRNP. **(A)** Generation of COX41-defective HEK 293T cells. **(B)** The levels of COX41 in COX41-defective HEK 293T cells were determined by western blot assay. **(C)** Growth kinetics of the H5N1 (MOI, 0.01) in wild-type (WT) cells, COX41-KO-1 cells, and COX41-KO-1 cells with transfection of Flag-COX41. The supernatants in HEK293T cells were collected at 12, 24, 36, 48, and 72 h postinfection and subjected to viral titer determination by TCID_50_ assays. Data are mean ± s.d. (*n* = 3). ^***^p < 0.001. **(D)** WT and KO-1 cells were infected with H5N1 (MOI, 5 for 12 h.p.i. and MOI = 0.01 for 36 h.p.i.) after 1 h of adsorption. After infection, subcellular localization of NP was detected using a confocal microscope. Mean fluorescence intensity associated with NP expression level was analyzed by Image J software. Data are mean ± s.d. (*n* = 3 ~ 5). ^*^p < 0.05; ^***^p < 0.001.

To investigate how the cellular COX41 affects the replication of H5N1, we further used CRISPR/Cas9 technology to construct HEK 293T cell lines incapable of expressing functional COX41. In our amplified target fragments, a 4-base deletion at position 442 of COX41 in the KO-1 cell line and a 3-base mutation at position 466 of COX41 in the KO-2 cell line, both of which were induced by CRISPR/Cas9 and led to a codon shift in the cellular *COX41* gene (Figure 2A). This resulted in a frameshift deletion and ensured the expression deficiency of any functionally relevant domain. Compared with wild-type (WT) cells, COX41 was barely detectable in COX41-KO-1 cells and was expressed at very low levels in COX41-KO-2 cells (Figure 2B). Therefore, COX41-KO-1 cells were used for further experiments. Consistent with the gene knockdown assay, the growth kinetics of virus was decreased approximately 200-fold in COX41-KO-1 cells compared with that of virus in WT cells (Figure 2C). As expected, transfection of plasmids expressing COX41 rescued the defects of viral replication in COX41-KO-1 cells.

To further determine the effects of COX41 on vRNP nuclear transport, the subcellular localization analysis of viral nucleocapsid protein (NP), a structural protein that encapsidates the vRNP, was performed in COX41-KO-1 cells. As shown in Figure 2D, the viral NP proteins were distributed in both nuclei and cytoplasm at the single cycle and almost located in the cytoplasm at the muti-replication cycle, but it was retained in the nuclei of KO-COX41 cells at either single and muti-replication cycle (Zhang et al., 2016). Collectively, these results suggest that COX41 plays an important role in the replication of H5N1, as well as in the nuclear export of vRNP.

### Lycorine attenuates COX41 accumulation *in vitro*

Our previous studies have demonstrated that lycorine inhibited H5N1 infection through blocking the nuclear export of vRNP (He et al., 2013). However, the molecular target for lycorine against H5N1 is still unclear. Considering the important roles of COX41 in vRNP transport, we next investigated whether lycorine inhibits vRNP transport by suppressing the expression of cellular COX41.

**Figure 3:**
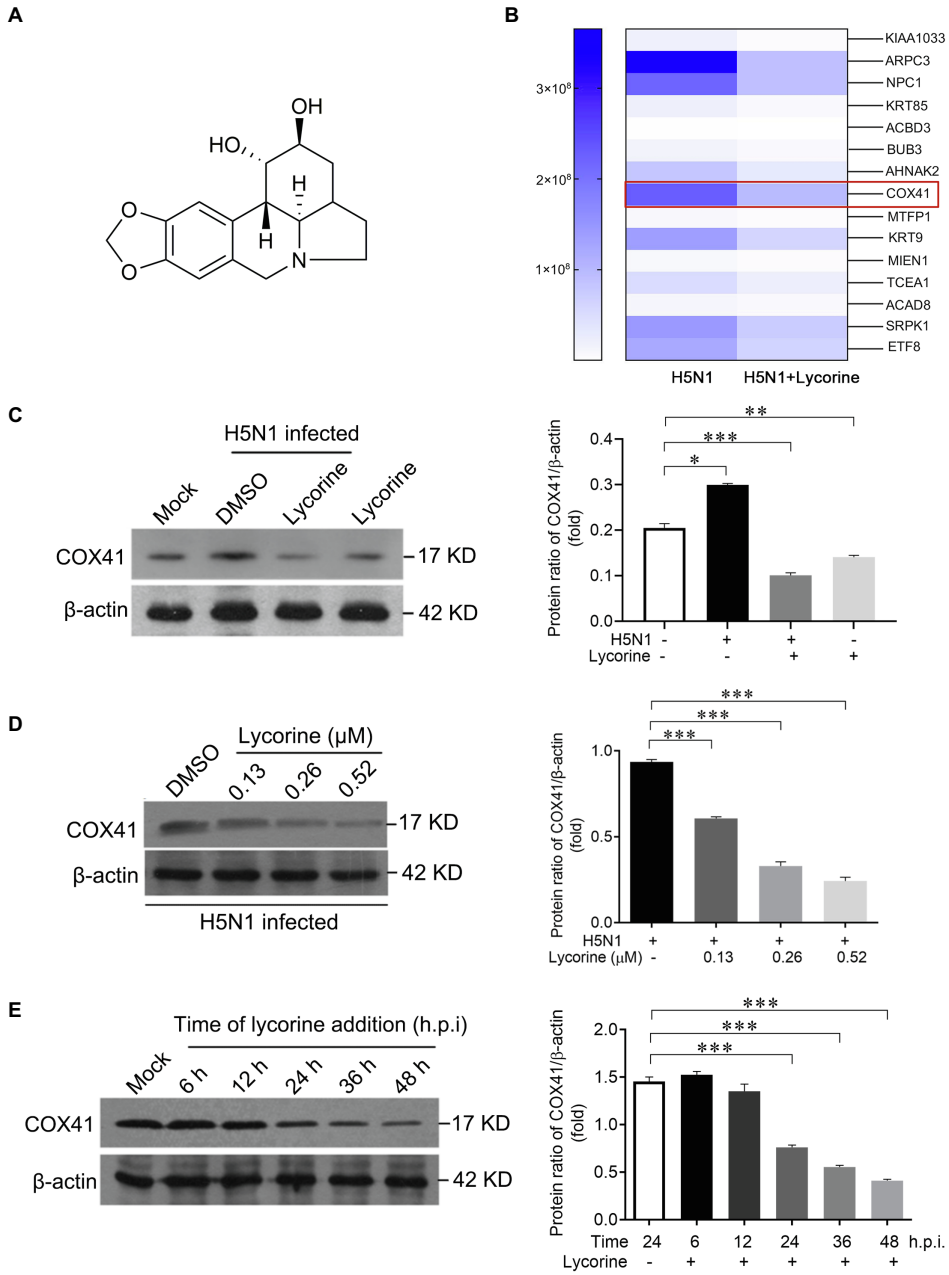
The expression of COX41 was inhibited by lycorine treatment *in vitro*. **(A)** Chemical structure of lycorine. **(B)** Quantitative proteomic analysis of H5N1-infected cells in responses to lycorine treatment. MDCK cells were infected with H5N1(MOI, 1) in the presence of lycorine (0.52 μM) for 12 h. The upregulated proteins in H5N1-infected cells but downregulated proteins after lycorine treatment are presented as a heat map. **(C)** Western blot and quantitative analysis of COX41 levels in H5N1-infected MDCK cells. MDCK cells were infected with H5N1 (MOI, 0.01) and treated with DMSO or lycorine (0.52 μM). The level of COX41 in cells was determined at 24 h after the viral infection. Data are mean ± s.d. (*n =* 2 ~ 3). ^*^p < 0.05, ^**^p < 0.01, ^***^p < 0.001 **(D)** Western blot analysis and quantitative analysis the expression of COX41 in H5N1-infected cells in the presence of DMSO or different concentrations of lycorine. Data are mean ± s.d. (*n* = 2 ~ 3). ^***^p < 0.001. **(E)** Lycorine suppressed the expression of COX41. Plasmids expressing FLAG-tagged COX41 were transfected into HEK 293 T cells and incubated for 4 h, followed by treatment of lycorine (0.52 μM). The cell lysates were harvested at 6, 12, 24, 36, and 48 h after transfection and analyzed by western blot assay. Data are mean ± s.d. (*n* = 2 ~ 3). ^***^p < 0.001.

We first performed a SILAC-based proteomic approach to detect the differentially expressed proteins between H5N1-infected cells and H5N1-infected cells in the presence of lycorine. Importantly, the expression level of COX41 and viral titers in cells were potently suppressed after the treatment of lycorine (Figures 3A,B; Supplementary Figure S1), consistent with the results of the immunoblotting assay (Figure 3C). Next, we determined that lycorine inhibited the expression of COX41 in a concentration-dependent manner. The expression levels of COX41 in the virus-infected cells were dramatically suppressed by lycorine at the tested concentrations without exhibiting substantial cytotoxicity (Figure 3D and Supplementary Figure S2). Furthermore, the results of time-of-addition assay indicated that lycorine significantly inhibited the expression of COX41 until multiple viral replication cycles (Figure 3E). These results, combined with our previous studies, suggest that lycorine inhibits H5N1 infection and the nuclear export of vRNP by suppressing the expression of COX41.

### Lycorine attenuates COX41-induced ROS production and modulates ERK activity

**Figure 4:**
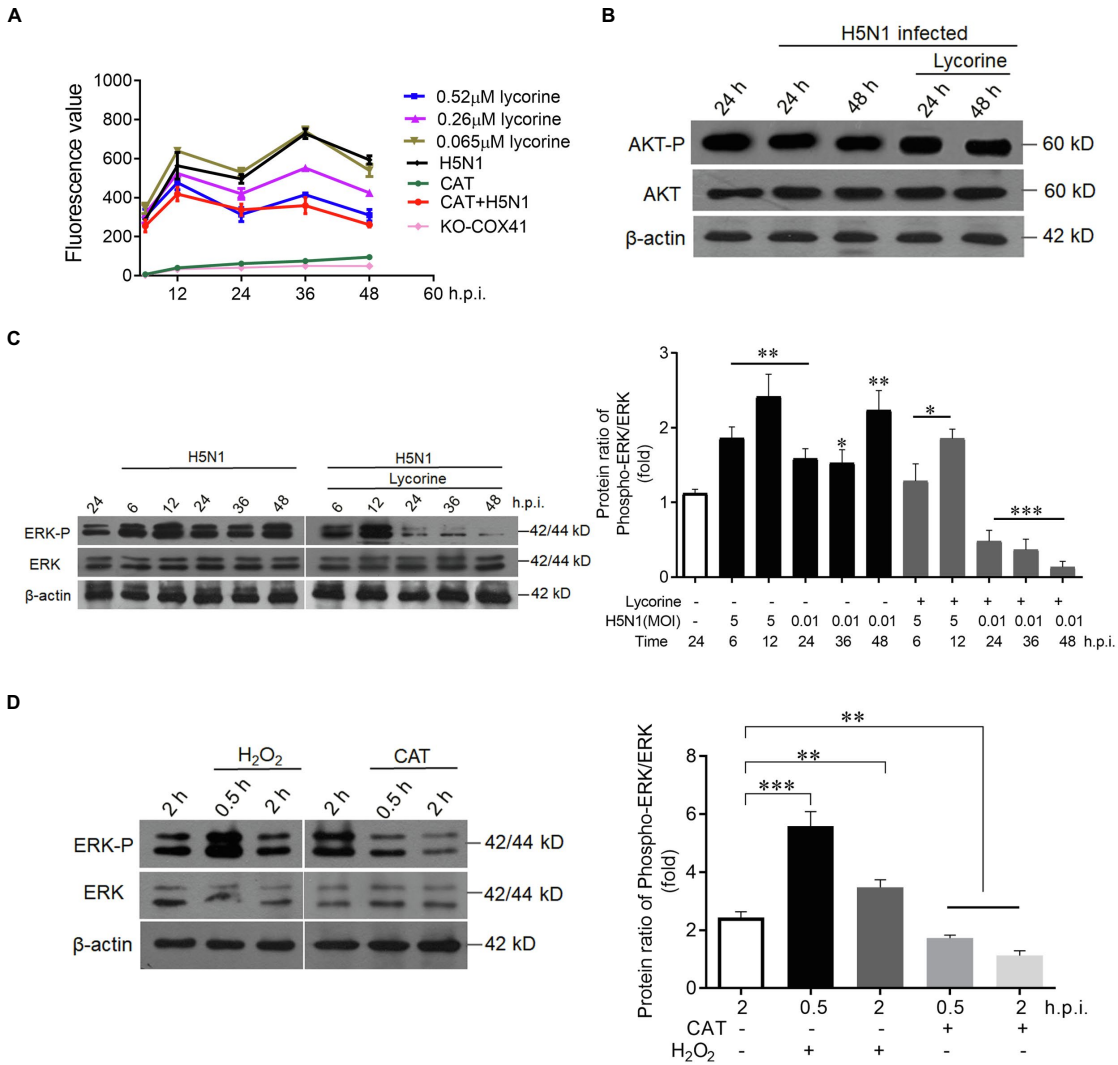
Lycorine inhibited the expression of COX41 through the ROS-ERK1/2 axis. **(A)** Lycorine suppressed the production of H5N1-induced ROS in wild-type cells or COX41-KO-1 cells. Catalase (CAT, 1 mg/ml). Data are mean ± s.d. (*n* = 3). **(B)** Lycorine did not affect PI3K/Akt signaling in MDCK cells that were treated with lycorine (0.52 μM) at the indicated time points after the viral infection. **(C)** Western blot and quantitative analysis of the inhibitory effect of lycorine on phosphorylation levels of ERK. MDCK cells were infected with H5N1 in the absence or presence of lycorine (0.52 μM). The protein levels were detected at indicated time points postinfection. Data are mean ± s.d. (*n* = 3). ^*^p < 0.05; ^**^p < 0.01; ^***^p < 0.01. **(D)** ROS increased phosphorylation levels of ERK. H_2_O_2_ (1 mm, a ROS activator) and CAT (1 mg ml^−1^, a ROS inhibitor) were used to test the phosphorylation of ERK. Data are mean ± s.d. (*n* = 3). ^*^p < 0.05; ^**^p < 0.01; ^***^p < 0.01.

We next investigated how COX41 located in the cytoplasm affected the transport of vRNP in the nucleus. Previous studies have reported that COX41 is involved in ROS production (Pajuelo Reguera et al., 2020). Therefore, we first examined whether lycorine reduced the expression of ROS in H5N1-infected cells. As shown in Figure 4A, the ROS levels were increased after viral infection as expected, it was potently suppressed in the cells that were treated with lycorine or catalase (CAT), a ROS scavenger.

Next, we determined the effects of lycorine on PI3K/Akt and Raf/MEK/ERK pathways, both of which can regulate the ROS production and facilitate vRNP export from the nucleus to the cytoplasm (Vlahos et al., 2011; Zhou et al., 2015; Zhang et al., 2016). As shown in Figure 4B, lycorine at 0.52 μM did not affect PI3K/Akt signaling. In contrast, the H5N1-stimulated phosphorylation of ERK (P-ERK) was dramatically reduced in the cells that were treated with lycorine at the same concentration (Figure 4C). The phosphorylation levels of ERK were significantly increased at 0.5 h within the activator of ROS (H_2_O_2_) and sharply decreased at 0.5 and 2 h within the inhibitor of ROS (CAT; Figure 4D). Overall, these results support that lycorine effectively inhibits the expression of COX41, decreases ROS production, and leads to downregulating the phosphorylation of ERK, thus resulting in the retention of vRNP in the nucleus.

### Lycorine protects mice against lethal AIV challenge

**Figure 5:**
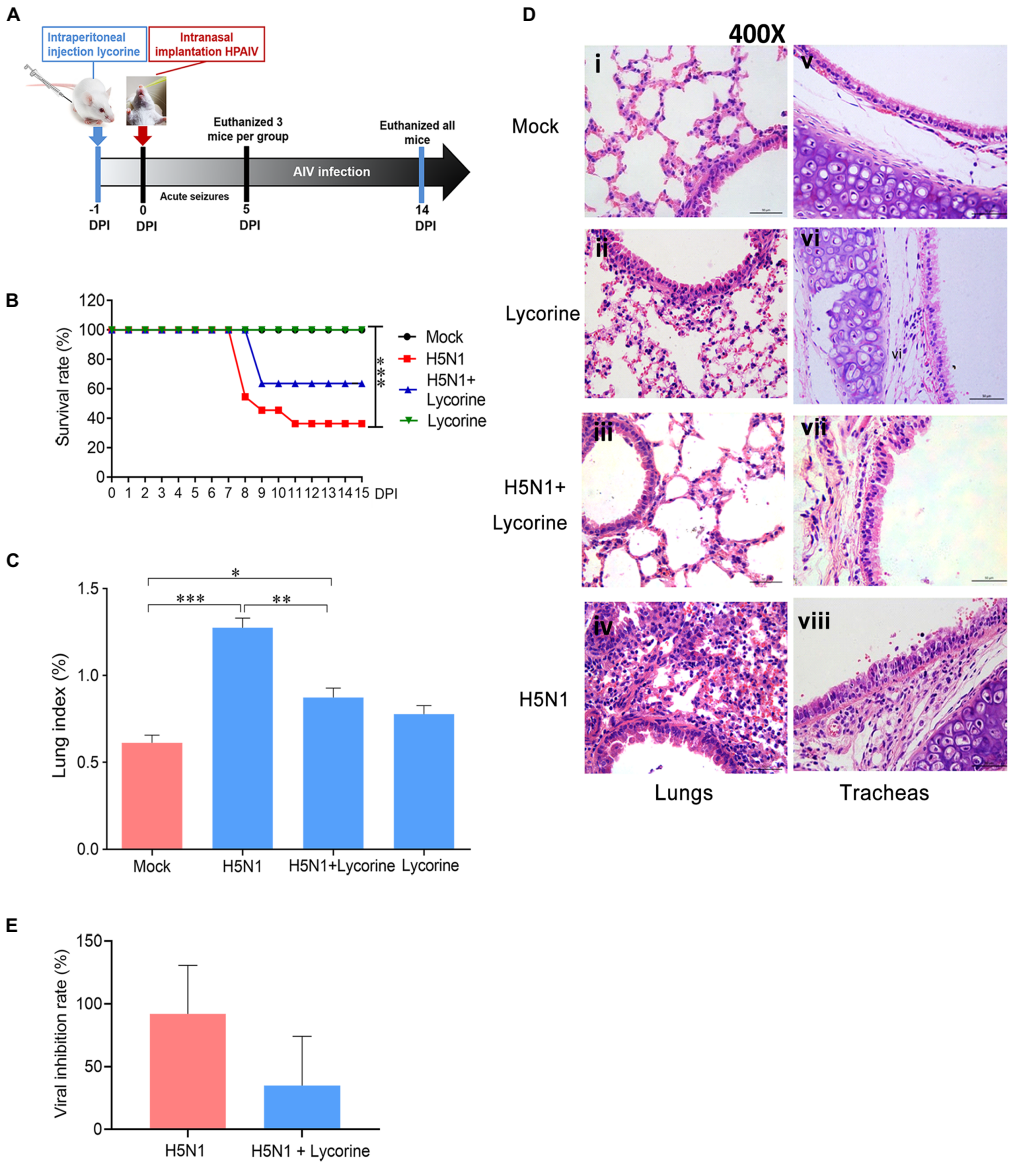
Anti-influenza effects of lycorine on the survival rate and lung index, pathological damage, and viral titers in lungs of mice. **(A)** *In vivo* schedule. The BALB/c mice (*n* = 11) underwent i.p. injection with 5 mg kg^−1^ day^−1^ of lycorine at 24 h before infection with 2LD_50_ virus and administered for 16 consecutive days. The mice from each group were randomly selected and euthanized on day 5 or 14. **(B)** Lycorine treatment increased the survival rate of the H5N1-infected mice. **(C)** Lycorine attenuated the lung indices, which were stimulated by H5N1 infection. Lung index = lung weight (g)/body weight (g) × 100. **(D)** Representative H&E staining images of lung and trachea tissues are shown as 400× magnification. **(E)** Viral titers in lung tissues of mice as determined by TCID_50_ assay. Data are mean ± s.d. (*n* = 5). ^*^p < 0.05; ^**^p < 0.01; ^***^p < 0.001.

To determine whether lycorine is protective against influenza-infected mice, we firstly tested the toxicity of lycorine in animal models and estimated the 50% LD_50_ of lycorine on H5N1 (GD178 strain)-infected mice (Supplementary Figure S3). Our results indicated that lycorine treatment had little effect on the mouse body weight, which was consistent with previously reported data, in which lycorine exhibited low toxicity to mice at a curative dose (Lamoral-Theys et al., 2009). As shown in Figure 5A, the anti-influenza activity of lycorine was evaluated according to the *in vivo* schedule. Mice from each group were randomly selected and euthanized on day 5 to assess the survival rate (Figure 5B), lung index (Figure 5C), pathological analysis (Figure 5D), and viral titers (Figure 5E). As expected, neither morbidity nor mortality was observed in the mock group and lycorine controls, and the weight loss trends were also consistent. By contrast, GD178-infected mice exhibited signs of illness by 3 ~ 4 dpi, which manifested as weight loss, lethargy, piloerection, and hunched posture, with generalized cachexia caused by rapidly fatal infection at 7–8 dpi and a mortality rate of over 64% (Figure 5B). The clinical symptoms of the lycorine-treated mice after viral infection were better than those of the virus-challenged control group, which became weak and died at 9 dpi. Consistent with these findings, the beneficial effects of lycorine, lower clinical symptoms and maintenance of body weight, largely increase the survival rate of the H5N1-infected mice.

Previous studies have shown that low lung indices correlate well with protection against influenza virus infection (Yang et al., 2013). As shown in Figure 5C, the lung indices of the lycorine-treated group were lower than those of the untreated mice after challenge with the H5N1 virus, and the lung viral titers were attenuated in the lycorine-treated group compared with those in the virus-infected group (Figure 5E).

We subsequently determined the efficacy of lycorine treatment through histopathological examinations. No obvious histopathological changes were observed in the lung and trachea sections from the mock group (Figures 5Di,v). The AIV infection group exhibited significant pathological changes, including interstitial pneumonia, multifocal bronchopneumonia, extensive hyperemia (Figure 5Div), exfoliation of the tracheal respiratory epithelium, and destruction of ciliated epithelia (Figure 5Dviii). Pathological changes were ameliorated in the lycorine-treated group. Lung and trachea sections were structurally intact with slight hyperemia and edema (Figures 5Dii,vi). Similar histopathological observations were observed in the viral infection and lycorine treatment groups (Figures 5Diii,vii). Collectively, these results demonstrate that low doses of lycorine could attenuate acute organ injury caused by H5N1 infection.

## Discussion

Most of the current drugs are used to treat HPAIV infection by directly targeting viral factors (e.g., neuraminidase protein). In the past decades, these direct-acting drugs have made significant contributions to reducing HPAIV transmission around the world. However, a variety of issues, such as drug resistance and adverse effects, have been developed over time. One strategy to reduce the drug-resistance risk is to develop inhibitors targeting host cell factors involved in viral replication. This study demonstrated that inhibition of cellular COX41, cytochrome C oxidase (CcO) subunit 4 isoform 1, could serve as a new therapeutic option for H5N1 infection.

COX41 is an isoform of CcO subunit 4 and associated with several human diseases, such as cancers and mitochondrial disorders (Oliva et al., 2017). However, the effects of COX41 on viral infection have been rarely reported. In this study, several lines of evidence suggest that COX41 plays an important role in H5N1 replication. First, the level of COX41 in H5N1-infected cells was upregulated as compared with non-infected cells. Second, the overexpression of COX41 promoted the viral replication. The viral titers in COX41-transfected cells were increased by 2.5-fold at 72 h postinfection. Third, knockdown the expression of COX41 has dramatically suppressed the replication of H5N1, as indicated by lower viral titers in cells transfected with COX41 siRNA. Meanwhile, the knockout of COX41 gene in cells retained the vRNP in the nucleus. Previous studies have reported that several subunits of CcO were involved in the viral pathogenesis such as herpes simplex viruses and coxsackieviruses (Saffran et al., 2007; Xu et al., 2011), but the molecular mechanism of these Cco subunits on the viral pathogenesis is still elusive. Moreover, it is currently unclear the roles of COX41 during influenza virus infection. This study provided the direct evidence that the replication of H5N1 is positively correlated with the cellular COX41 level.

**Figure 6:**
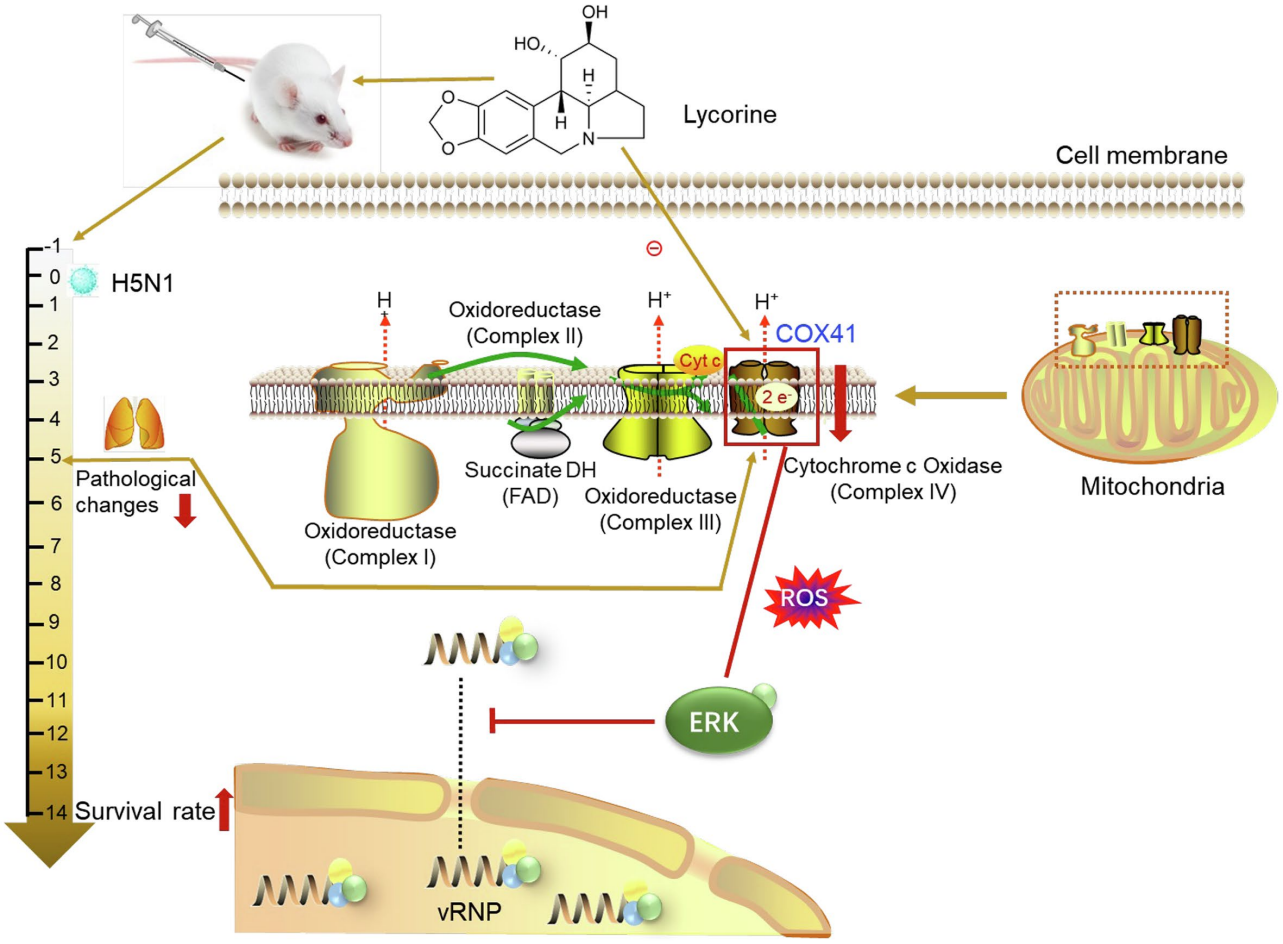
Protective effects of lycorine on H5N1 infection *in vivo* and *in vitro*. COX41 activates the ERK signaling and promotes the nuclear export of vRNP. Lycorine inhibits the nuclear export of vRNP and viral replication through suppressing the expression of COX41.

Furthermore, we investigated the possibility of suppressing the expression of COX41 as an antiviral approach through a series of *in vitro* studies. Lycorine, a small-molecule compound isolated from Amaryllidaceae plants, has been identified to reduce the levels of COX41 in several types of cells and inhibit the infection of H5N1 in this study. Although we did not elucidate the molecular target of lycorine on the viral infection, our results suggested that the compound suppressed the expression of COX41, leading to blockage of nuclear export of vRNP and inhibition of viral replication (Figure 6). According to some previous reports, lycorine exerts antiviral activity by targeting the RNA-dependent RNA polymerase (RdRp) or viral protease (Guo et al., 2016; Chen et al., 2020). However, these proposed mechanisms inadequately explain the broad-spectrum antiviral activities of lycorine because RdRp and viral protease are not ubiquitous factors for different viruses. By contrast, COX41 is expressed in the mitochondria of most mammalian cells and has been identified as a pro-viral factor for H5N1 replication in this study. Further studies on investigating the roles of COX41 in other viral infections are needed and will help us to assess the feasibility of COX41 as a potential target for the development of broad-spectrum antiviral drugs.

Currently, three antiviral drugs approved by the US Food and Drug Administration (FDA) are recommended to treat influenza virus infection: Rapivab (peramivir), Relenza (zanamivir), and Tamiflu (oseltamivir), all of which are neuraminidase (NA) inhibitors (Shie and Fang, 2019). However, the drug resistance caused by these inhibitors is becoming more and more serious (Long et al., 2019). Moreover, most of the drug candidates against the influenza virus continue to be virus-targeted inhibitors, which would inevitably result in resistance problems due to the high mutation rate of the viral genes (Krammer et al., 2018; Yin et al., 2021). Search for inhibitors targeting host cell proteins is becoming an alternative strategy to reduce the resistance risk. This is because the eukaryotic cell genes are highly conserved and have a lower mutation risk than viral genes, particularly the genes of RNA viruses (e.g., H5N1). The present study indicated that lycorine not only improved the health status of mice with respiratory symptoms but also strongly attenuated the lesions in the lungs caused by the H5N1 infection. Furthermore, we did not obtain the resistant virus after culturing H5N1 in cells with the continuous treatment of lycorine (Supplementary Figure S4). These findings suggest that inhibition of cellular COX41 might be a viable approach to reduce the influenza virus infection with low resistance risk.

We note that CcO plays a key role in the regulation of cellular energy metabolism. Deficiency of CcO may lead to several human diseases such as disorder of mental function, hypertrophic cardiomyopathy, and movement problems. It is important to carry out further studies to investigate whether inhibitors of CcO are associated with these side effects *in vivo*, although we did not observe apparent toxic effects of lycorine in cells, as well as in mice at the tested dosages in this study. Future studies including safety evaluation and pharmacokinetic characteristics *in vivo* are needed, which is important for assessing the potential value of lycorine as a lead compound for anti-influenza drug development.

## Conclusion

This study indicates that the replication of H5N1 in cells is positively correlated with the cellular COX41 level. Suppressing COX41 expression in the host cell by lycorine resulted in the blockage of the nuclear export of vRNP and inhibition of the replication of H5N1. Treatment with lycorine could attenuate viral load and acute organ injury in the lung of mice that were infected with H5N1. These findings suggest that COX41 is a positive regulator of H5N1 replication and represents a potential target for anti-influenza drug development.

## Data availability statement

The datasets presented in this study can be found in online repositories. The names of the repository/repositories and accession number(s) can be found at: ProteomeXchange Consortium—PXD031293.

## Ethics statement

The animal study was reviewed and approved by Institutional Animal Care and Use Committee of South China Agricultural University (No. 2016020).

## Author contributions

JH, LW, HH, and YL conceived and designed the experiments. HH, JH, BL, HL, and YZ performed the experiments. JH, HH, LW, WT, and BL analyzed the data. JH wrote the manuscript. WT, LW, WQ, and WY revised the manuscript. All authors contributed to the article and approved the submitted version.

## Funding

This work was financially supported by the National Natural Science Foundation of China (grant no. 81822042), the Basic and Applied Basic Research Fund of Guangdong Province (grant nos. 2021A1515010822, 2020B1515120066, and 2019A1515011994), and the National Natural Science Foundation of China (grant nos. 81603165, 81973190, and 82003610).

## Conflict of interest

The authors declare that the research was conducted in the absence of any commercial or financial relationships that could be construed as a potential conflict of interest.

## Publisher’s note

All claims expressed in this article are solely those of the authors and do not necessarily represent those of their affiliated organizations, or those of the publisher, the editors and the reviewers. Any product that may be evaluated in this article, or claim that may be made by its manufacturer, is not guaranteed or endorsed by the publisher.

## Supplementary material

The Supplementary material for this article can be found online at: https://www.frontiersin.org/articles/10.3389/fmicb.2022.862205/full#supplementary-material

SUPPLEMENTARY FIGURE S1 Inhibition of the plaque-forming ability of H5N1 by lycorine. 1,000 PFU of H5N1 (GD178 strain) was incubated with lycorine or DMSO. At 48 h after infection, the viral titers in cells were measured by plaque assay.

SUPPLEMENTARY FIGURE S2 The cytotoxicity of lycorine on MDCK cells as determined by CCK-8 assay. Data are mean ± s.d. (*n* = 6).

SUPPLEMENTARY FIGURE S3 Body weight changes of mice inoculated with PBS (control), DMSO in PBS (Mock), or lycorine (5 mg kg^−1^) in PBS. Mice were monitored for body weight loss throughout the observation period for 16 days. Data are mean ± s.d. (*n* = 7).

SUPPLEMENTARY FIGURE S4 H5N1 (GD178) was grown in MDCK cells in the presence of lycorine (0.46 μm). Drug susceptibilities of the virus at passages 10, 20, and 30 were determined by CCK-8 assay.

## Footnotes

^1^ https://www.addgene.org/Feng_Zhang/

## Abbreviations

HPAIV: Highly pathogenic avian influenza virus
SILAC: Stable-isotope labeling with amino acids
vRNP: Viral ribonucleoprotein
AIV: Influenza A virus
MOI: Multiplicity of infection
COX41: Cytochrome c oxidase subunit 4 isoform 1
HA: Hemagglutinin
NA: Neuraminidase
i.p.: Intraperitoneal injection
h.p.i.: Hours postinfection
