# Supplementary figures and images for: Identification of cytochrome c oxidase subunit 4 isoform 1 as a positive regulator of influenza virus replication
Source: Front Microbiol. 2022 Jul 19;13:862205. doi: 10.3389/fmicb.2022.862205 (PMC9343726; doi:10.3389/fmicb.2022.862205)

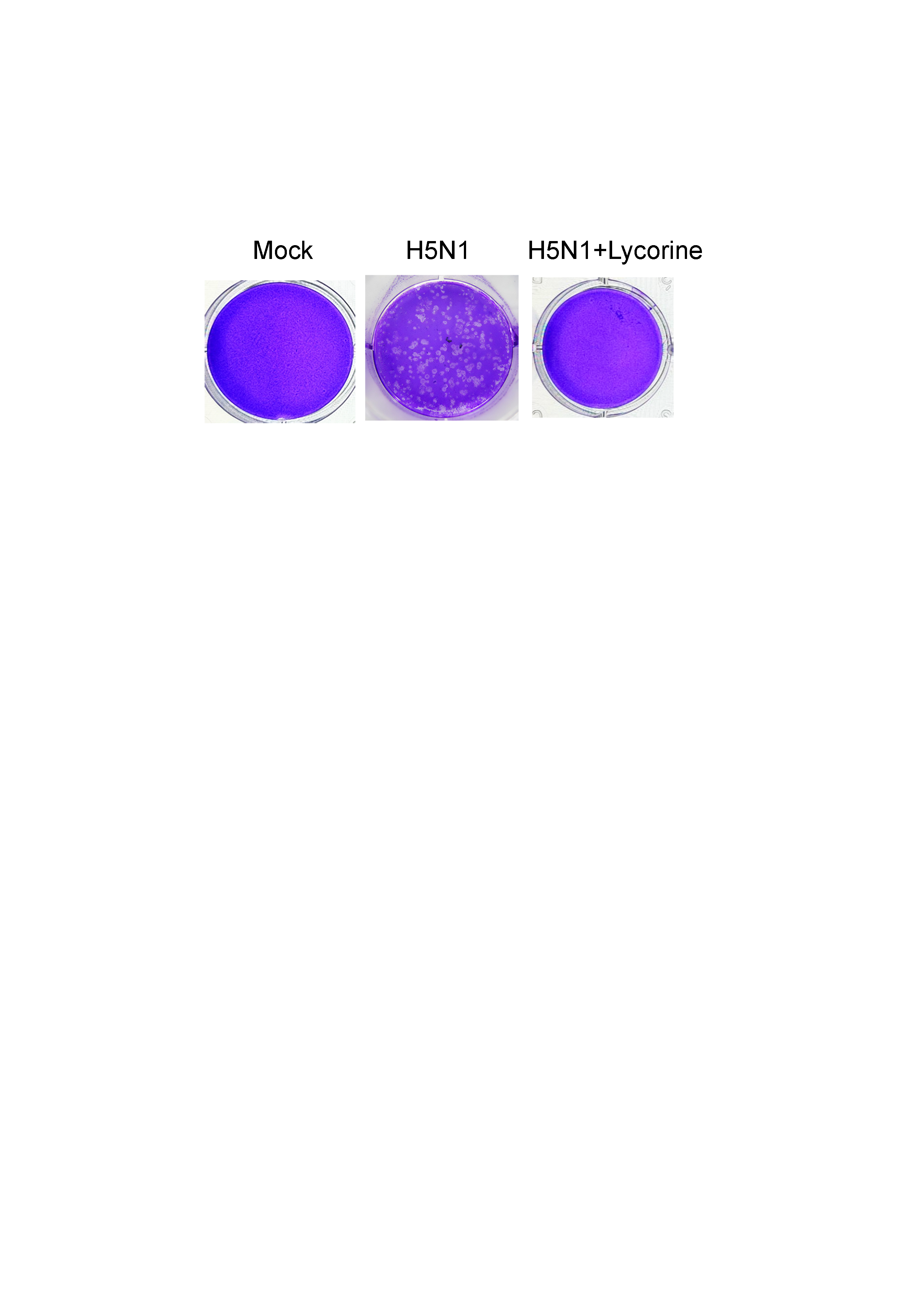

Supplement: SUPPLEMENTARY FIGURE S1 — Inhibition of the plaque-forming ability of H5N1 by lycorine. 1,000 PFU of H5N1 (GD178 strain) was incubated with lycorine or DMSO. At 48 h after infection, the viral titers in cells were measured by plaque assay. [file Image_1.TIF]

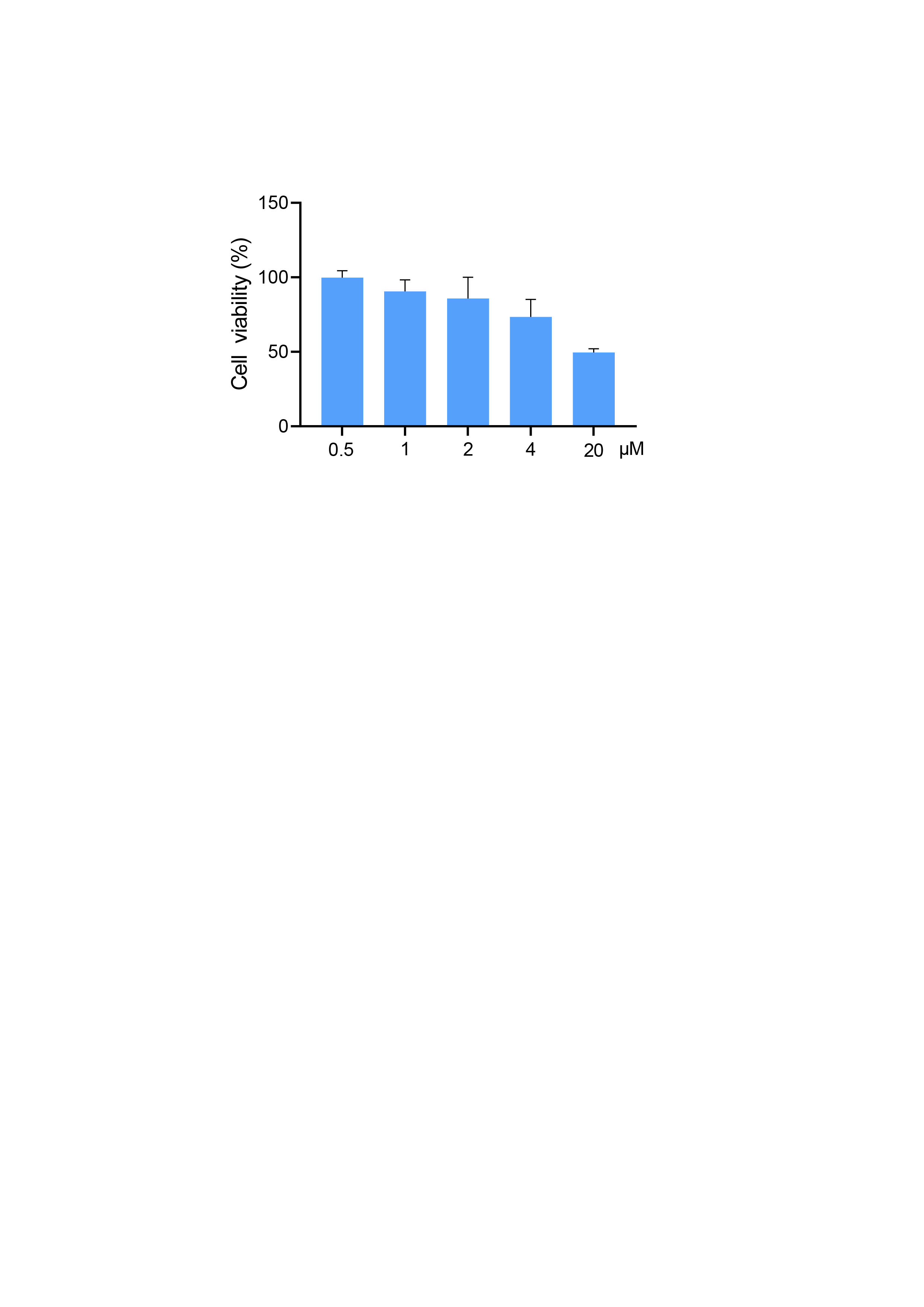

Supplement: SUPPLEMENTARY FIGURE S2 — The cytotoxicity of lycorine on MDCK cells as determined by CCK-8 assay. Data are mean ± s.d. (n = 6). [file Image_2.TIF]

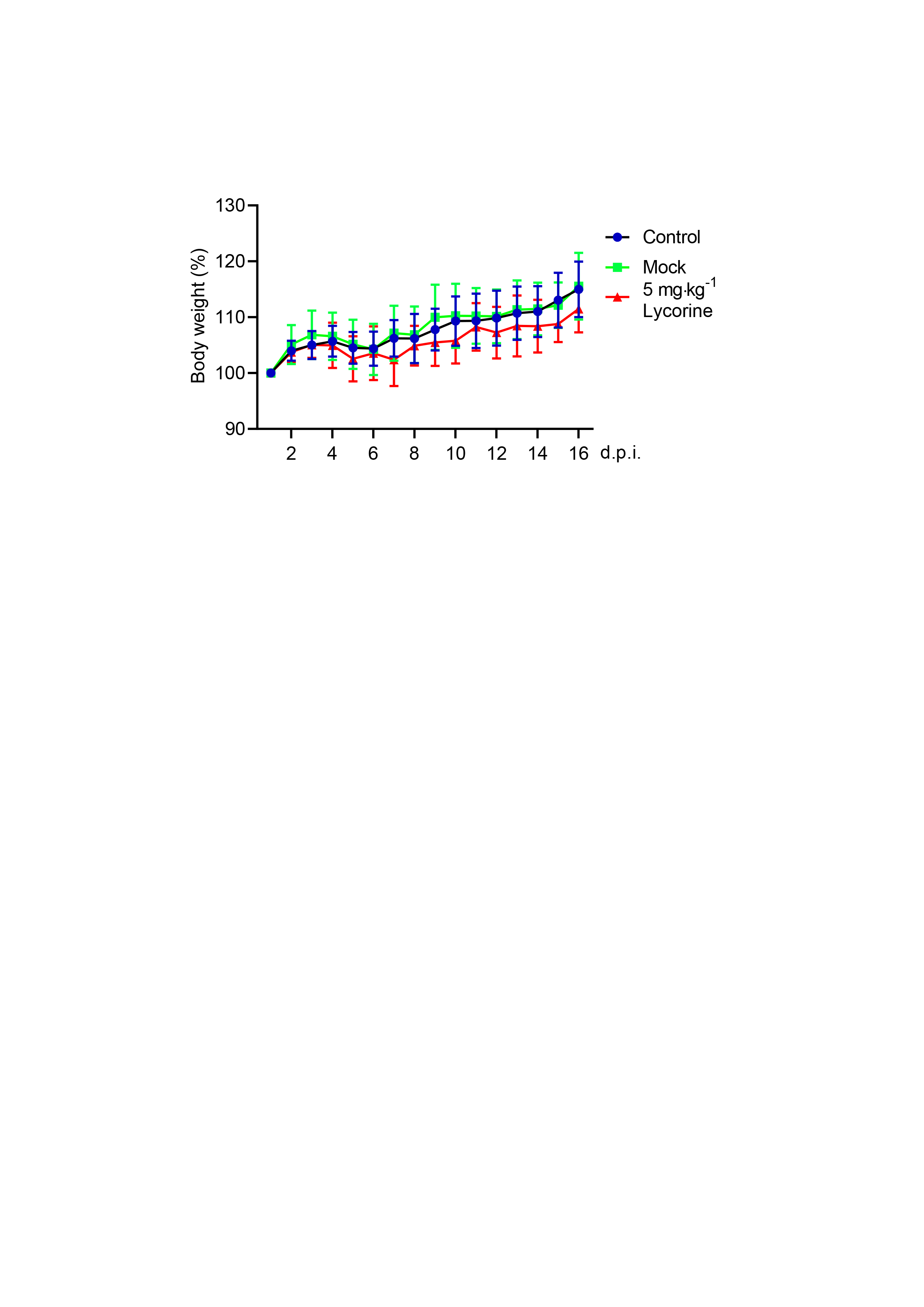

Supplement: SUPPLEMENTARY FIGURE S3 — Body weight changes of mice inoculated with PBS (control), DMSO in PBS (Mock), or lycorine (5 mg kg−1) in PBS. Mice were monitored for body weight loss throughout the observation period for 16 days. Data are mean ± s.d. (n = 7). [file Image_3.TIF]

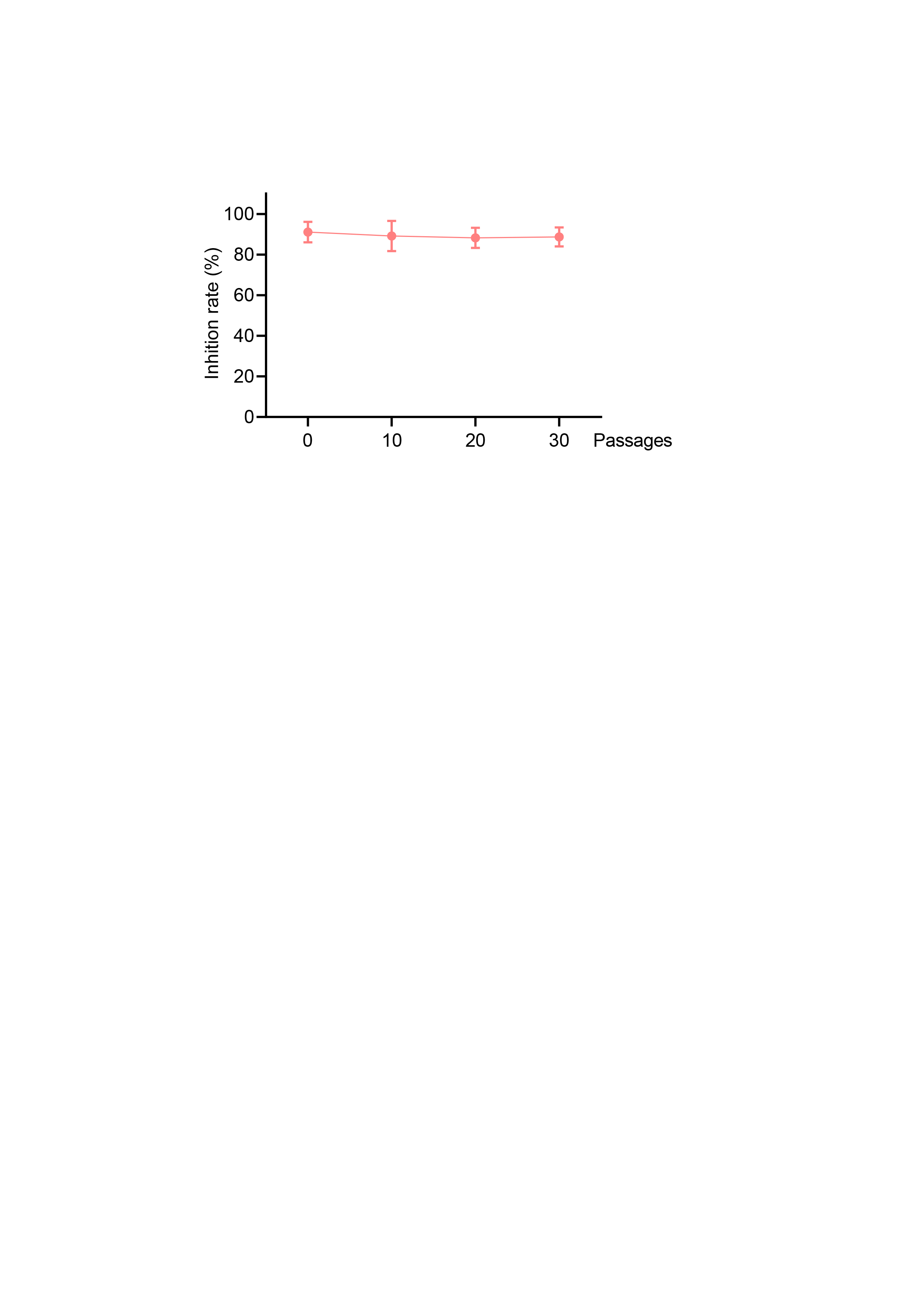

Supplement: SUPPLEMENTARY FIGURE S4 — H5N1 (GD178) was grown in MDCK cells in the presence of lycorine (0.46 μm). Drug susceptibilities of the virus at passages 10, 20, and 30 were determined by CCK-8 assay. [file Image_4.TIF]
